# Stem Cell Transplantation for Peripheral Nerve Regeneration: Current Options and Opportunities

**DOI:** 10.3390/ijms18010094

**Published:** 2017-01-05

**Authors:** Liangfu Jiang, Salazar Jones, Xiaofeng Jia

**Affiliations:** 1 Department of Orthopaedics, The Second Affiliated Hospital and Yuying Children’s Hospital of Wenzhou Medical University, Wenzhou 325027, China; john94007@163.com; 2 Department of Neurosurgery, University of Maryland School of Medicine, Baltimore, MD 21201, USA; SAJones@som.umaryland.edu; 3 Department of Orthopaedics, University of Maryland School of Medicine, Baltimore, MD 21201, USA; 4 Department of Anatomy and Neurobiology, University of Maryland School of Medicine, Baltimore, MD 21201, USA; 5 Department of Biomedical Engineering, The Johns Hopkins University School of Medicine, Baltimore, MD 21205, USA; 6 Department of Anesthesiology and Critical Care Medicine, The Johns Hopkins University School of Medicine, Baltimore, MD 21205, USA

**Keywords:** peripheral nerve, regeneration, stem cells, transplantation

## Abstract

Peripheral nerve regeneration is a complicated process highlighted by Wallerian degeneration, axonal sprouting, and remyelination. Schwann cells play an integral role in multiple facets of nerve regeneration but obtaining Schwann cells for cell-based therapy is limited by the invasive nature of harvesting and donor site morbidity. Stem cell transplantation for peripheral nerve regeneration offers an alternative cell-based therapy with several regenerative benefits. Stem cells have the potential to differentiate into Schwann-like cells that recruit macrophages for removal of cellular debris. They also can secrete neurotrophic factors to promote axonal growth, and remyelination. Currently, various types of stem cell sources are being investigated for their application to peripheral nerve regeneration. This review highlights studies involving the stem cell types, the mechanisms of their action, methods of delivery to the injury site, and relevant pre-clinical or clinical data. The purpose of this article is to review the current point of view on the application of stem cell based strategy for peripheral nerve regeneration.

## 1. Introduction

Peripheral nerve injuries (PNI) are mainly related to trauma, tumor, and iatrogenic lesions, leading to neurologic deficits and functional disability. The incidence of PNI is estimated at about 18 per 100,000 persons every year in developed countries, whereas it is relatively higher in developing countries [1,2].

Primary repair with suture is the preferred management for nerve discontinuities without a gap. Despite an excellent tension-free nerve repair, the functional outcome can be limited by inflammation, scar formation, and misdirection of regenerating sensory and motor axons. Regeneration is still subject to a rate of approximately 1 mm/day [3]. For nerve discontinuities with a gap, nerve autografts are useful but limited by availability and donor site morbidity. The various synthetic conduits and acellular allografts on the market, which we have previously reviewed, are not generally recommended for gaps >3 cm [4]. Although advanced bioengineering can recreate the nerve extracellular matrix, nerve conduits lack the critical cellular component, specifically Schwann cells (SC) critical for regeneration. SCs, by secreting various neurotrophic and neurotropic factors, develop a microenvironment conducive to axonal regeneration [5]. SCs interact with the surrounding extracellular matrix to stabilize myelin in the normal state, and can switch to a pro-myelination phenotype during regeneration [6].

Multiple neurotrophic factors including nerve growth factor (NGF) and glial-cell-derived neurotrophic factors (GDNFs) are stimulated by nerve injury and accelerate axon growth [7]. However, mature SCs in peripheral nerve do not maintain a growth-permissive phenotype to support axonal regeneration. Moreover, the requirement of sufficient SCs within a short time seriously limits its clinical application [8]. Stem cells are of interest as a source of Schwann-like cells that would take residence in the nerve and support a stable pro-regeneration environment.

The aim of this article is to discuss the features of different types of stem cells relevant to peripheral nerve regeneration, their mechanism of benefits, cell delivery, and relevant pre-clinical or clinical data of each.

## 2. Stem Cell Sources

Stem cells refer to cells that possess the capability of self-renewal in addition to differentiation to a more specialized cell type [1]. According to the development stage, stem cells can be divided into embryonic stem cells and adult stem cells. Stem cells can be characterized by their differentiation potential. Totipotent stem cells can form an entire embryo including the extraembryonic tissues. Pluripotent stem cells can trigger the mesoderm, endoderm, and ectoderm. Postnatal or adult stem cells are capable of multi-lineage differentiation in cells of only one germ layer. Unipotent or progenitor stem cells can only differentiate into one defined cell type [2]. The differentiation potential of stem cells can be related to their developmental stage. Differentiation potential decreases from an embryonic stem cell to a specialized tissue stem cell. Fully differentiated adult somatic cells do not naturally have any differentiation potential. Induced pluripotent stem cells (iPSC) are a type of pluripotent stem cell that can be generated directly from adult cells [3]. Thomson et al. showed that somatic cells could be transcriptionally regulated to express a more embryonic phenotype, thus creating the first induced pluripotent stem cells (iPSC) [1].

This review evaluates different types of stem cells based on development stage including iPSC and tissue source.

### 2.1. Embryonic Stem Cells (ESCs)

ESCs are pluripotent stem cells derived from the blastocyst stage of embryonic development [4]. ESCs can differentiate into somatic cells from all three embryonic germ layers. Several strategies with ESCs have been employed in the area of peripheral nerve injuries.

To replace the necessary Schwann cells needed for nerve regeneration, Ziegler et al. developed a protocol to generate Schwann cells from human ESCs with 60% efficiency [5]. The differentiated Schwann cells were shown to associate with axons. In a rat sciatic nerve injury model Cui et al. achieved significantly improved regeneration by the microinjection of neutrally-induced ESCs [6]. Immunostaining demonstrated that the ESCs survived and had differentiated into Schwann-like cells [6]. An alternative strategy is to inject the ESCs into the target muscle at the time of nerve injury/repair to prevent muscle denervation changes and slightly speed recovery [7].

ESCs are also of interest for the generation of additional stem cell lines. Adult stem cell lines typically require an invasive procedure for harvesting and can be limited by the quantity obtained. Mesenchymal stem cells (MSCs) can be generated from ESCs and have been used in pre-clinical animal models [8,9].

ESCs have great potential, but are not without their disadvantages. ESCs have the potential for teratoma formation [4]. In addition, there are limited sources of human embryos from which ESCs are obtained. There also exists the ethical dilemma of using a human embryo which contains the potential to form a complete individual for research or clinical applications.

### 2.2. Neural Stem Cells (NSCs)

NSCs are stem cells capable of differentiating into neurons or glial cells. They are present during neurogenesis for the proper organization of the brain and spinal cord. NSCs have been isolated from murine models and proliferated in vitro [10,11]. In the adult human brain, NSCs take residence in the subventricular zone and hippocampus [12,13]. Adult NSCs are thought to have a limited role in central nervous system injury [14]. In 1992, two groups reported the successful isolation of NSCs from the brain tissue of adult mice [10,11]. A variety of studies have demonstrated that NSC implantation is beneficial in both acute and chronic PNI [15,16]. However, NSCs have several disadvantages and limitations. Commercial murine C17.2 NSCs showed a high rate of neuroblastoma formation in an animal model [17]. Despite NSCs being discovered in multiple areas in the brain, they are difficult to harvest from the brain [18]. In addition, directed differentiation of specialized neural cell lines is difficult and the current methods are only effective in limited cases [19].

#### 2.2.1. Mesenchymal Stem Cells (MSCs)

Though initially identified as a multipotent fibroblastic cell population within bone marrow different from a hematopoietic lineage [20], MSCs can be obtained from a wide range of non-marrow sources. MSCs have been isolated from adipose tissue, peripheral blood, amniotic fluid, umbilical cord, tendon and ligaments, hair follicle, synovial membranes, olfactory mucosa, dental pulp, and fetal tissue [21]. MSCs are of considerable interest in tissue regeneration given their differentiation potential, easy isolation, and immunomodulation [22]. MSCs are inherently capable of differentiating into all mesoderm lineages: fat, bone, muscle, and cartilage [22]. Under the proper environment, MSCs differentiation can be guided into non-mesenchymal lineages, such as neurons, astrocytes, and Schwann-like cells [23] to support nerve regeneration. The sub-types of MSCs based on tissue source and related application in PNI are discussed.

##### Bone Marrow-Derived Stem Cells (BMSCs)

BMSCs can differentiate into neurons, astrocytes, and SC-like cells under suitable conditions [23]. The fate of the BMSCs may be dictated by post transplantation physiological microenvironment. Almost 5% of BMSCs were induced to differentiate into Schwann cells within the lesioned nerve tissue 33 days after transplantation [24]. Nijhuis et al. showed that BMSCs implanted within a muscle in vein autograft led to an early increase in nerve growth factor and S100 positive Schwann-like cells compared to muscle in vein autograft alone in a rat sciatic nerve injury model [25]. Wang et al. demonstrated superior recovery with BMSCs suspended in matrix compared to autologous nerve graft in a 10-mm rabbit sciatic nerve injury model [26]. Rabbits with BMSCs suspended in matrix had significantly greater motor nerve conduction velocities and amplitudes [26]. Interestingly, the regenerative benefits of BMSCs plated onto poly-caprolactone filaments were superior to exogenous Schwann cells plated onto filaments in a rat model [27]. Raheja et al. showed that BMSCs improve in a dose-dependent manner the extent of myelination, thickness of myelin, and axonal thickness in a rat model [28]. There is no clinical data regarding the beneficial effects of BMSC transplantation for nerve regeneration, however, it has already been clinically used to treat myocardial infarction [29,30] and spinal cord injury [31].

Although BMSCs present more easily harvested than ESCs and NSCs, the capacity of proliferation and differentiation of BMSCs is inferior to the latter. In addition, BMSCs are limited by the need for an invasive procedure for autologous harvesting. The procurement procedures are invasive and painful that usually need anesthesia, whereas the obtained stem cell fraction is obviously lower than from other sources.

##### Adipose-Derived Stem Cells (ADSCs)

ADSCs can be derived from adipose tissue obtained from common procedures such as liposuction. These cells are particularly advantageous since they are available via minimally invasive harvesting with a high cellular yield of (0.25−0.375) × 10^6^ cells per milliliter of liquid fat after 4 to 6 days in culture with medium containing 10% fetal bovine serum [32]. They show higher proportion and superior proliferation and differentiation potential compared with BMSCs [33]. ADSCs can be differentiated into an SC-like phenotype (differentiated adipose-derived stem cell, dASC) which shares morphological and functional properties with SC, thus representing a valid SC alternative [34,35,36,37]. Several studies have indicated there were no significant difference for sciatic nerve regeneration by using 2- or 14-day dASCs [38,39]. Liu et al. cut rat sciatic nerves into 1-cm fragments, and then soaked them in a filtered differentiation-inducing culture medium for two days. Differentiated rat ADSCs were similar to genuine Schwann cells after being incubated with the above induction medium for five days. The vast majority of studies show an augmented effect of ADSCs seeded in silicone conduits on peripheral nerve regeneration [40,41]. Particularly, ADSC transplantation decreases muscular atrophy, facilitates sorting of axons and myelination, and reduces inflammation [42,43]. Some investigators consider ADSCs to have a similar therapeutic effect compared with autologous SCs and BMSCs [44]. Rather than differentiate to SC phenotype, it is hypothesized that ADSCs mainly facilitate endogenous SC recruitment by releasing growth factors such as NGF, vascular endothelial growth factor (VEGF), and brain-derived neurotrophic factor (BDNF) [39,45,46] for nerve protection and regeneration, as the therapeutic effect is maintained for several weeks even after many ADSCs are gone [47]. ADSCs may aid angiogenesis both by direct differentiation into vascular endothelium, and their associated paracrine effects [48,49]. Like BMSCs, the neurotrophic potential of ADSCs is influenced by the harvest site [50], fat layer [51], and donor age [46]. Another restriction is the differentiation potential towards adipocytes, which is unfavorable for nerve regeneration [52]. Accessible harvest and better stem cell characteristics make ADSCs one of the optimal choices for pre-clinical studies.

#### 2.2.2. Fetal-Derived Stem Cells

Fetal tissues are the most primitive source of MSCs and have received less genetic damage caused by age, environment, and disease [53]. Stem cells can be derived from multiple sources, such as amniotic fluid, amniotic membrane, umbilical cord, and Wharton’s jelly. Since such tissues are generally abandoned after birth, fetal-derived stem cells are in sufficient excess and can be easily obtained without the need for invasive procedures. The cells obtained can proliferate in culture and differentiate into a neural phenotype [54].

##### Amniotic Tissue-Derived Stem Cells (ATDSCs)

ATDSCs are derived from amniotic fluid or the amniotic membrane. ATDSCs possess the characteristics of both mesenchymal and NSCs [55] and can differentiate into neural tissue [56]. They also exhibit strong angiogenic potential, as their implantation augmented blood perfusion and enhanced intraneural vascularity in addition to promote peripheral nerve regeneration [57,58]. Survival of ATDSCs following transplantation is a challenge to their clinical application. Genetic modification and inhibition of inflammatory mediators can restrain the apoptotic cascade [59]. Several reports have explored the effect of gene mutation in ATDSCs on PNI. Human ATDSCs with GDNF modification significantly enhance viability, regeneration, and motor function in animal models [60]. Stromal cell-derived factor-1α (SDF-1α) expression in muscle and nerve after PNI can recruit ATDSCs for their deposition, thus in time, ATDSC injection at high levels of SDF-1α effectively increases the number of ATDSCs at the repair site, promoting nerve regeneration [61].

##### Umbilical Cord-Derived MSCs (UC-MSCs)

UC-MSCs are a promising candidate for cell therapies because of their differentiation and proliferation potential. They are easily accessible from the postnatal tissue that is discarded after birth, thus facing fewer ethical problems. Though UC-MSCs have the proliferative ability, there are few reports about the tumorigenesis of UC-MSCs or UC-MSC-derived cells in transplantation experiments [62]. Matsuse et al. reported a system to induce UC-MSCs to differentiate into cells with SC properties using β-mercaptoethanol followed by retinoic acid and a set of specific cytokines [63]. Further investigation revealed that Schwann-like cells differentiated from UC-MSCs generated neurotrophic factors like NGF and BDNF [64]. In addition, the differentiated human Schwann-like cells transplanted into rat transected sciatic nerve under immunosuppression maintained the differentiated phenotype, elicited axonal regeneration from the proximal segment, and constructed peripheral nerve system (PNS) tissue. This was even functionally equivalent to authentic SCs based on walking track analysis [65]. This indicates that UC-MSCs could be used to alternatively generate Schwann-like cells for PNI regenerative therapy.

##### Wharton’s Jelly MSCs (WJMSCs)

Wharton’s jelly is a special primitive connective tissue protecting vessels in the umbilical cord [66]. Cells in its stromal compartment show specific mesenchymal features, thus named Wharton’s jelly MSCs (WJMSCs) [67]. WJMSCs have shown the capacity to differentiate to Schwann-like cells. Furthermore, they can generate neurotrophic factors including NGF, BDNF, and neurotrophin-3 (NT-3), and trigger axon growth in vitro [68]. Thus, Wharton’s jelly can become an ideal source of MSCs, characterized as unique and easily accessible.

Fetal tissue provides a prospective alternative for stem cells acquisition. The main obstacles of their application, alloreactivity and immunoreactivity, may not be encountered in stem cells from other sources. Cell bank for the storage of fetal products provides a resolution for this conundrum.

#### 2.2.3. Skin-Derived Precursor Stem Cells (SKP-SCs)

SKP-SCs located in the dermis are an available source for somatic multipotent cells. In addition to durable proliferative ability, SKP-SCs can differentiate to a diverse array of cell types, including melanocytes, craniofacial cartilage, bone, connective tissue, vascular smooth muscle, endocrine cells, neurons, and glial cells [69]. SKP-SCs cultured in neuregulin-1β express the same markers with SCs [70]. Moreover, both undifferentiated and differentiated SKP-SCs have exhibited acceleration on nerve regeneration. SKP-SCs treatment significantly increases mean axon counts and reduces the percentage of myelin debris [71]. Several studies demonstrated the superior outcomes of SKP-SCs on de-myelination and crush injury [70,72], and acute and chronic transection injury [71].

#### 2.2.4. Hair Follicle Stem Cells (HFSCs)

Hair follicle stem cells are embryologically from the neural crest, and are an abundant and accessible source for pluripotent stem cells [73]. HFSCs are readily expanded in culture but cannot be kept for long periods, which is similar to SKP-SCs. ESC transcription factors Nanog, Oct4, and nestin are positively expressed in HFSCs. Furthermore, HFSCs also can differentiate to a variety of cell types, such as adipocytes, smooth muscle cells, melanocytes, neurons, and glial cells [74]. One of the advantages of HFSCs is that they can differentiate into pure human SC population rapidly in a straightforward way, without the requirement of genetic manipulation. Undifferentiated HFSCs used in a murine model with sciatic and tibial nerve crush and transection injuries demonstrated significantly improved function [75]. Improved outcomes in 4-cm rat sciatic nerve defects were seen by the addition of neurons and Schwann cells derived from HFSCs to an acellular xenograft [76].

#### 2.2.5. Dental Pulp Stem Cells (DPSCs)

New odontoblast formation and dentin production in response to severe tooth damage suggested the existence of MSCs in dental pulp tissue. DPSCs were first isolated in 2000 and found to differentiate into odontoblast-like cells [77]. They also exhibit the feature of MSCs that can be induced into multi-lineage including neural cells under appropriate culture condition. Specifically, DPSCs can express neural markers, generate neurotrophic factors, promote axon guidance, and differentiate into functionally active neurons [78]. Although available data is limited, DPSCs have been shown to chemoattract trigeminal ganglion axons [79], differentiate into SCs or nourish SC to support dorsal root ganglion neurite outgrowth, and guide myelin repair [80,81]. DPSCs secrete various trophic factors that enhance peripheral nerve regeneration [82]. Moreover, DPSCs are reported to have a stronger proliferation and greater clonogenic potential, and a larger stem/progenitor cell population in comparison to BMSCs [83], suggesting their clinical applicability. Moreover, they were reported to improve function through combination with a pulsed electromagnetic field in the form of SC-like cells [84]. In a manner similar to fetal tissue, autologous cells can be easily harvested but require storage [82]. Cell banking should thus be considered due to the properties of easy isolation and cryopreservation.

#### 2.2.6. Muscle-Derived Stem/Progenitor Cells (MDSPCs)

MDSPCs can be derived from skeletal muscle and have sustained self-renewal, long-term proliferation, and multipotent differentiation [85,86]. Although MDSPCs have shown potential for regeneration of skeletal and cardiac muscles, bone, and articular cartilage, there is limited research about their role in human nerve repair. Some researchers reported that MDSPC transplantation could be applied for neuropathy as they can differentiate into SCs, perineurial/endoneurial cells, vascular endothelial cells, and pericytes needed for neurovascular regeneration [87,88]. Peripheral nerve damage frequently accompanies musculoskeletal trauma. MDPCs from traumatized muscle tissue secrete the neurotrophic factors that are associated with muscle tissue reinnervation [89].Though MDSPCs present an opportunity in peripheral nerve regeneration together with muscle atrophy prevention, limited evidence and the appropriate harvest site are still challenges in the current stage.

### 2.3. Induced Pluripotential Stem Cells (iPSCs)

Considering the limitation of various types of stem cells, researchers tried to artificially induce the stem cells. Takahashi demonstrated a protocol of defined transcription factors to induce pluripotency in mouse and human fibroblasts [3]. The ability of reprograming cells supplies new hope to develop an individual-specific pluripotent stem cell that can overcome the restriction of ESCs. At present, understanding of iPSCs has advanced in multiple disease mechanisms and they are used for in vitro drug screening and therapeutic efficacy evaluation [90]. In addition to differentiation into somatic cells, the method of inducing iPSCs differentiation along neural lineages has been established [91]. In spite of subdued efficiency and enhanced variability during the differentiation process [92], iPSCs have presented a regenerative potential in animal models of central and peripheral nerve injury [93].

iPSCs have been used to induce neurospheres in 3-D-culture to maintain the ability to form neural or glia cells [94]. iPSCs have been applied to coat a tissue-engineered bioabsorbable nerve conduit and implanted to PNI mice. Axonal regeneration and myelination were enhanced without teratoma formation following 48-week observation, suggesting their alternative application potential in PNI [95].

Though iPSCs are favorable over ESC given the avoidance of ethical issues and need for immunosuppression, there exists still for iPSCs in clinical applications such as epigenetic memory from the original somatic cells, chromosomal aberrations, and tumorigenicity [96].

The comparison of stem cells from different resources is listed in Table 1. For the clinical application of stem cell-based transplantation, the ideal source should be individualized, immune tolerant, easy to harvest, non-tumorigenic, able to be integrated in the host nerve tissue, and efficient in replacement.

## 3. Mechanism of Action

**Figure 1:**
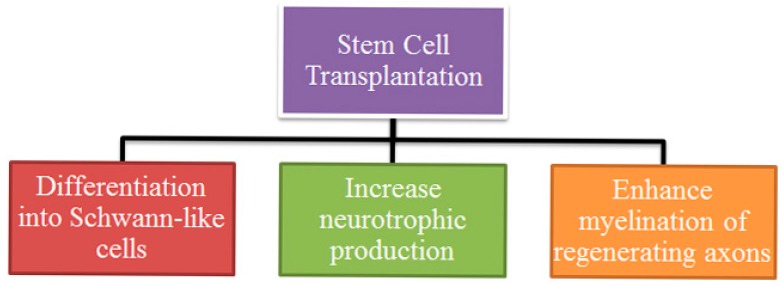
Mechanism of stem cell transplantation for peripheral nerve injury (PNI) regeneration.

The impact of stem cells transplantation in PNI mainly depends on their capacity in differentiation phenotype, ability in enhancing neurotrophic action, and promotion of myelin formation (Figure 1).

### 3.1. Differentiation Type of Stem Cells

The self-renewal capacity of stem cells makes it possible to deliver numerous cleavage cells to the damage site [23]. The stem cells continue proliferating after migrating to the injured nerve tissue, and further differentiate to the necessary cell type under the appropriate microenvironmental conditions [97]. It is confirmed that NSCs can be induced to a peripheral neuron, SC, or smooth muscle phenotype upon co-culture with cells from the nervous system. Furthermore, about 5% of BMSCs can spontaneously transdifferentiate into SCs without specific intervention [24]. However, the differentiation rate of naive precursor cells in the peripheral nerve is relatively low [58]. Predifferentiating stem cells toward a desired phenotype in vitro by chemical induction, biological treatment, gene transfection, or co-culture with neural cells before injection is an effective method. The representative protocol of MSC induction is exposure to or transfection by growth factors β-mercaptoethanol (β-ME) and alltransretinoic acid (RA), the cytokines forskolin (FSK), basic fibroblast growth factor (bFGF), and platelet-derived growth factor (PDGF) sequentially [98]. In particular, BMSCs can express NSC markers by transfecting the transmembrane region and intracellular domain of notch [99] or differentiating into neurosphere cells upon Noggin transfection [100]. Stem cells were maintained in differentiation medium for 2 weeks in most protocols [101,102]. This is time consuming. Finally, SC-like cells must be co-cultured with dorsal root ganglion neurons to maintain stable morphological features upon juxtacrine neuronal cues [103].

With predifferentiation, SC markers are increased and maintained for longer time upon treatment before delivery [70]. After differentiated stem cell transplantation, accelerated transected axons regenerate and achieve better remyelinization [104]. The extent of recovery was comparable to or even greater than that observed after Schwann cell transplantation [105]. Other experiments showed primary Schwann cells were significantly improved with respect to distal stump sprouting compared to differentiated bone marrow-derived mesenchymal stem cells (dMSC) and dASC-loaded conduits [106]. In contrast, some scholars reported that predifferentiation facilitates post-transplant cell death, which may be caused by enhanced ability of major histocompatibility complex antigens or reduced proliferation ability compared with naïve stem cells [107]. Another potential drawback of MSCs is the tumorigenic capability, as shown by the high rate of tumorigenesis observed in rat sciatic nerve injury model transplanted by C17.2 neural stem cells [17].

### 3.2. Neurotrophic Action Enhancement

Other than differentiation to appropriate cells, stem cells also provide a beneficial microenvironment for neural cell survival and neurogenesis by secreting bioactive neurotrophic molecules [46,108]. In addition to support SC differentiation, maturation, and proliferation, stem cells may exhibit better performance in enhancing neurotrophic action. MSCs synthesize and release a variety of growth factors, such as nerve growth factor (NGF), brain-derived neurotrophic factor (BDNF), GDNF, neurotrophin-3 (NT-3), VEGF, and ciliary-derived neurotrophic factor (CDNF) [109]. SKP-SCs increase BDNF, NGF, and NT-3 compared with single SCs in culture [108]. ADSCs also upregulate protein expression of BDNF, glial growth factor, neuregulin-1, VEGF, HGF, and insulin-like growth factor [46]. Furthermore, overexpressed neurotrophic factors facilitate the regeneration of peripheral nerves even beyond the nerve injured region. ADSCs may alleviate dorsal root ganglion loss upon inhibiting caspase-3 activity in a neurotrophin-dependent manner [110].

The level of growth factors in the microenvironment also affects the influence of transplanted stem cells for feedback. NGF neutralizing antibody can abrogate the stimulatory effect of BMSCs on neurite growth of sensory and sympathetic neurons in vitro [111]. BDNF neutralizing antibody reduces the influence of ADSCs on nerve sprouts growth in vivo [112].

### 3.3. Myelin Promotion

Myelination is another major factor that determines the regeneration quality and functional recovery in PNI. Multiple types of somatic stems cells present the ability to myelinate neuronal cells in the form of SC-like cells in vitro [113]. SCs play a critical role for myelin sheath structure and function recover by synthesizing a large amount of myelin proteins, such as myelin basic protein (MBP), P0, and PMP22 [114]. Similar to SCs, stem cells differentiated into SC-like cells also show the capacity of supporting myelination in regenerated nerves in vivo [113]. A study SC-like BMSCs injected to the autologous vein conduits significantly increase the number of myelinated axons and improve the facial nerve functional recovery through enhancing myelin factors mRNA expression [104]. Transplantation of gingiva-derived mesenchymal stem cells (GMSCs) and induced neural progenitor cells (iNPCs) promotes peripheral nerve repair/regeneration, possibly by promoting remyelination of Schwann cells mediated via the regulation of the antagonistic myelination regulators, c-Jun and Krox-20/EGR2 [115].

## 4. Stem Cell Delivery

Stem cells can be delivered through numerous ways (Table 2). The stem cells can be suspended in a medium that can be directly microinjected into the nerve ending [116]. The process of microinjection can be traumatic both to the stem cells and delicate intra-neural architecture, leading to abnormal cell distribution. Another method is to suspend the stem cells in fibrin matrix and inject the matrix around the repair sites [26,117]. In repairs with a conduit, stem cells can be injected in the conduit lumen or on the conduit matrix. Tse et al. describes a method for inkjet printing Schwann cells with phenotypic analysis over seven days. Glial cell viabilities of >90% were detected immediately after printing [118]. Three-dimensional printing [119,120] aims at creating tissues with multiple cell types within a scaffold for mimicking native tissue, which is a progressive step towards peripheral nerve printing. Further refinement of the delivery system may provide better cell distribution and improve efficacy. Three-dimensional printing technology for fabricating can provide the desired geometry, such as multichannel, bifurcating and personalized structures, which allows the customization of a nerve guidance conduit (NGC) that precisely matches a particular nerve defect of a patient [121,122].

Natural conduits such as vein and artery grafts are abundant in extracellular matrix (ECM) proteins such as collagen and laminin, thus contributing to cell adhesion and axonal guidance [25]. Commercially natural conduits are usually filled with ECM components including collagen [126] and fibrin [127]. Artificial conduits are mainly synthetized by polyglycolic acid [123], silk fibroin [124], poly-epsilon-caprolactone [27], polyhydroxybutyrate [130], silicone tube [131], polytetrafluoroethylene [125], or chitosan [128]. Recently, biological and nanofibrous conduits have rapidly developed, while the concern for their application in cell therapy include degradation waste and velocity [132]. Natural materials are prone to degrade in a non-toxic manner, and the velocity might be too fast. In contrast, part of synthetic polymers can produce acidic materials during degradation which is detrimental to the microenvironment and cellular activity [133]. The internal structure within the basal lamina is beneficial for axonal guidance compared with hollow lumen tubes, which are composed of organized multiple fibers [128,129] or less orderly collagen sponges [125].

## 5. Perspective

Peripheral nerve regeneration is a dynamic process. Stem cell transplantation still remains in the pre-clinical stage and has yet to make significant headways into clinical practice. In spite of genetic manipulation, cell instability, and tumorigenesis, stem cell homing and migration remains a concern. Simple application of stem cell transplantation has shown some improvements in outcomes, but is still inferior to nerve repair with conventional techniques. Pre-clinical and eventually clinical studies comparing different types of stem cell are needed. Other factors such as optimal Schwann cell differentiation, exact underlying mechanisms of action, and cell delivery have yet to be solidified, making it difficult to draw clear conclusions. Cell banks may provide benefits for future applications of stem cell therapy.

## Figures and Tables

**Table 1:** Comparison of stem cells from different sources in peripheral nerve regeneration. ESCs: embryonic stem cells; NSCs: neural stem cells; BMSCs: bone marrow-derived stem cells; ADSCs: adipose-derived stem cells; SKP-SCs: skin-derived precursor stem cells; HFSCs: hair follicle stem cells; DPSCs: dental pulp stem cells; MDSPCs: muscle-derived stem/progenitor cells; iPSCs: induced pluripotential stem cells; SCs: Schwann cells.

Stem Cell	Classification	Advantage	Disadvantage	Preclinical or Clinical Use	Mechanism
ESCs	Pluripotent stem cells	Homogenous, no detrimental impact of age and disease, unlimited cell number, better differentiation potential, and longer lasting proliferation capacity	Teratoma formation, ethical dilemma	Preclinical [8,9]	Myelination and/or neurotrophic factors
NSCs	Multipotent stem cells		Difficult to be harvested	Preclinical [15,16]	Replace Schwann cells
BMSCs	Multipotent cells	Easily accessible without ethical concerns	Lower capacity of proliferation and differentiation, invasive procedure for autologous harvesting	Preclinical [25,26]	Myelination, neurotrophic factors
ADSCs	Multipotent stem cells	Easy to harvest, higher proportion and superior proliferation	Differentiation potential towards adipocytes	Preclinical [40,41,42,43]	Myelination, neurotrophic factors, reduce inflammation
Fetal-derived stem cell	Multipotent stem cells	Less immunoreactivity	Cell bank for storage	Preclinical [57,58,65,68]	Augmented blood perfusion and enhanced intraneural vascularity
SKP-SCs	Multipotent cells	Easy to harvest	Long time to differentiate	Preclinical [71]	Replace Schwann cell myelination
HFSCs	Multipotent stem cells	Abundant and accessible source, differentiate into pure human SC population	Difficult to isolate	Preclinical [75]	Replace Schwann cell myelination, neurotrophic factors
DPSCs	Multipotent stem cells	Stronger harvesting and proliferation potential, as well as greater clonogenic potential	Require storage	Preclinical [80,81]	Replace Schwann cell myelination, neurotrophic factors
MDSPCs	Progenitor cells	Abundant and accessible source	Limited research	Preclinical [89]	Neurotrophic factors
iPSCs	Pluripotent stem cells	Inducible from easily obtainable somatic cells	Subdued efficiency and enhanced variability during the differentiation process, epigenetic memory from the original somatic cells, chromosomal aberrations, stronger tumorigenicity	Preclinical [93]	Replace Schwann cell myelination, neurotrophic factors

**Table 2:** Stem cells delivery in peripheral nerve regeneration.

Methods	Application	Advantage and Disadvantage	References
Micro injection		Traumatic both to the stem cells and delicate intra-neural architecture, abnormal cell distribution	Pang [116]
Conduit	Natural conduits or artificial	Difficult for cell delivery	Nijhuis [25] Costa [123] Yang [124] Carrier-Ruiz [27] Wakao [125]
Conduit + ECM	Collagen, fibirin	Good cell distribution, lack of 3-D construction	Pereira [126] di Summa [127]
Conduit + internal		Beneficial for axonal guidance	Wakao [125] Hu [128] Gu [129]
3-D print		Customization, good cell distribution	Weightman [121] Hu [122] Tse [118]
